# Pre-clinical and Clinical Implications of “Inside-Out” vs. “Outside-In” Paradigms in Multiple Sclerosis Etiopathogenesis

**DOI:** 10.3389/fncel.2020.599717

**Published:** 2020-10-27

**Authors:** Haley E. Titus, Yanan Chen, Joseph R. Podojil, Andrew P. Robinson, Roumen Balabanov, Brian Popko, Stephen D. Miller

**Affiliations:** ^1^Department of Microbiology-Immunology, Northwestern University Feinberg School of Medicine, Chicago, IL, United States; ^2^Department of Neurology, Northwestern University Feinberg School of Medicine, Chicago, IL, United States; ^3^Cour Pharmaceutical Development Company, Inc., Northbrook, IL, United States; ^4^Interdepartmental Immunobiology Center, Feinberg School of Medicine, Northwestern University, Chicago, IL, United States

**Keywords:** multiple sclerosis, etiopathogenesis, demyelination, autoimmunity, animal models

## Abstract

Multiple Sclerosis (MS) is an immune-mediated neurological disorder, characterized by central nervous system (CNS) inflammation, oligodendrocyte loss, demyelination, and axonal degeneration. Although autoimmunity, inflammatory demyelination and neurodegeneration underlie MS, the initiating event has yet to be clarified. Effective disease modifying therapies need to both regulate the immune system and promote restoration of neuronal function, including remyelination. The challenge in developing an effective long-lived therapy for MS requires that three disease-associated targets be addressed: (1) self-tolerance must be re-established to specifically inhibit the underlying myelin-directed autoimmune pathogenic mechanisms; (2) neurons must be protected from inflammatory injury and degeneration; (3) myelin repair must be engendered by stimulating oligodendrocyte progenitors to remyelinate CNS neuronal axons. The combined use of chronic and relapsing remitting experimental autoimmune encephalomyelitis (C-EAE, R-EAE) (“outside-in”) as well as progressive diphtheria toxin A chain (*DTA)* and cuprizone autoimmune encephalitis (CAE) (“inside-out”) mouse models allow for the investigation and specific targeting of all three of these MS-associated disease parameters. The “outside-in” EAE models initiated by myelin-specific autoreactive CD4^+^ T cells allow for the evaluation of both myelin-specific tolerance in the absence or presence of neuroprotective and/or remyelinating agents. The “inside-out” mouse models of secondary inflammatory demyelination are triggered by toxin-induced oligodendrocyte loss or subtle myelin damage, which allows evaluation of novel therapeutics that could promote remyelination and neuroprotection in the CNS. Overall, utilizing these complementary pre-clinical MS models will open new avenues for developing therapeutic interventions, tackling MS from the “outside-in” and/or “inside-out”.

## Introduction

Multiple Sclerosis presents most often in young adulthood and is chronic as most patients live with the disease for decades. Recent studies on prevalence uncovered that nearly a million people live with MS within the United States (Culpepper et al., 2019; Nelson et al., 2019; Wallin et al., 2019). Approximately 85% of patients present with the relapsing-remitting form of MS (RRMS) that involves episodes of neurological deficits followed by phases of recovery (Steinman, 2009). The disease often slowly converts to a secondary-progressive form of MS (SPMS) that shows significant and irreversible neurological impairment (Stadelmann, 2011). The primary-progressive form of MS (PPMS) appears in the remaining patients and results in rapid progressive neurological decline (Miller and Leary, 2007).

With advances in technology, in addition to the traditional family history portion of a patient’s medical record, further characterization of a patient’s predisposition to disease can be evaluated by genomic sequencing. For example, recent studies have identified over 200 genomic and proteomic anomalies prevalent in the MS patient population, all directly or indirectly linked to the immune system (International Multiple Sclerosis Genetics Consortium et al., 2013; Manconi et al., 2018; International Multiple Sclerosis Genetics Consortium, 2019; Kotelnikova et al., 2019). Clinical presentation of the disease as well as chronic neuropathology highlight the destructive nature of the interaction between the immune system and the CNS.

The “outside-in” hypothesis constitutes a primary pathogenesis of autoimmune inflammation followed by a secondary pathogenesis of myelin degradation (Table 1). The “inside-out” hypothesis is a primary pathogenesis of oligodendrocyte (OL) injury/myelin destabilization and a secondary pathogenesis due to activation of a reactive inflammatory response (Table 1). Experimental murine models can recapitulate patient clinical presentations and pathological changes including inflammatory demyelination, axonal pathology, and immune cell infiltration utilizing both “outside-in” immune mediated demyelination and the “inside-out” CNS demyelination/neurodegeneration driven models (Table 2).

**TABLE 1:** “Inside-out” and “Outside-in” hypotheses of MS pathophysiology.

	“Inside-Out” Hypothesis	“Outside-In” Hypothesis
Primary Pathogenesis	OL injury / Myelin destabilized	Autoimmune inflammation
Secondary Pathogenesis	Reactive inflammatory response/Further myelin degradation	Myelin degradation

**TABLE 2:** “Inside-out” and “Outside-in” disease model systems highlighted in this review.

	“Inside-Out” models			“Outside-In” models			
	Epsilon Toxin Model	Diphtheria Toxin A (DTA) Model	Cuprizone autoimmune encephalitis (CAE) Model	Chronic Experimental Autoimmune Encephalomyelitis (C-EAE) Model	Relapsing Remitting Experimental Autoimmune Encephalomyelitis (R-EAE) Model	Theiler’s Virus-Induced Demyelinating Disease	Japanese Macaque Encephalomyelitis (JME) Model
Model species	Mouse	Mouse	Mouse	Mouse	Mouse	Mouse	Macaque
Induction of disease model (in Adults)	Epsilon toxin, produced by type B and D strains of *Clostridium perfringens*, a spore-forming gram-positive bacterium	Timed genetic expression of diphtheria toxin (tamoxifen induced *PLPCreER*^T^ for targeting OL)	Cuprizone diet for 2 weeks, then inject CFA (SubQ) and Pertussis Toxin (IP)	MOG_35__–__55_ + CFA (SubQ) and Pertussis Toxin (IP)	PLP_139__–__151_ + CFA (SubQ)	Theiler’s Murine Encephalomyelitis Virus (TMEV) intracerebral infection	Japanese Macaque Rhadinovirus (JMRV), spontaneous or injected
Disease model trigger	Epsilon Toxin induced cytotoxicity (OL)	DTA induced cell death (OL), secondary MOG peptide immune response	Cuprizone destabilizes myelin, Citrullinated MBP drives immune response	MOG_35__–__55_ peptide immune response	PLP_139__–__151_ peptide immune response	Response to TMEV and subsequent spreading to PLP and MBP epitopes	JMRV infection, MBP peptide immune response
Disease model pathogenesis	OL cytotoxicity, triggers demyelination	OL ablation, triggers demyelination/remyelination, secondary immune response (respond to MOG peptide)	Myelin breakdown, secondary immune response (respond to MBP epitope)	Immune infiltration (respond to MOG peptide), secondary CNS degeneration	Immune infiltration (initially respond to PLP peptide, later to MBP), secondary CNS degeneration	Immune response to virus, release of myelin epitopes inducing autoimmune pathology, secondary CNS degeneration	Immune response to virus and infiltration (respond to MBP peptide), secondary CNS degeneration

Presently, there is no cure for MS and the long-term treatment of MS patients is based on disease-modifying therapies that either suppress or modulate the immune system, and symptomatic management. Ideally, the mechanism(s) of action for an efficacious therapy would function to specifically target the root cause of the immune and CNS dysfunction. First, the underlying autoimmunity must be mitigated through re-establishing self-tolerance (McCarthy et al., 2014; Luo et al., 2016; Pearson et al., 2017, 2019). Second, neurodegeneration must be mitigated to protect the remaining function of CNS neurons. Third, tissue repair within the CNS must restore oligodendrocyte insulation and myelin sheath formation around damaged axons (Rodgers et al., 2013). As the etiology of the disease is unknown, utilizing both “outside-in” and “inside-out” models for single selective immune regulatory and myelin repair therapy as well as combination therapy in pre-clinical trials are imperative for success in developing effective therapeutics in patient clinical trials. This review will focus on the “outside-in” models, “inside-out” models of MS, and the multi-directional feedback between the immune system and the CNS.

## “Outside-In” MS Models

The process of drug discovery, approval, and future patient use requires initial testing in experimental models that recapitulate hallmarks of the human disease state. Initial inflammatory demyelination in Multiple Sclerosis and subsequent neurodegeneration is a result of multi-directional feedback involving CNS resident cells (i.e., oligodendrocytes, neurons, and microglia) and infiltrating immune cells (i.e., autoreactive T cells and B cells, inflammatory monocytes, and macrophages) (McFarland and Martin, 2007; Bhat and Steinman, 2009; Weiner, 2009). While the primary etiology of MS remains unknown and is likely multi-determinant, the disease involves the activation of the peripheral adaptive immune system against CNS myelin epitopes. However, the triggering event that initiates this autoimmune response is not understood. Antigen presenting cells (APCs) (i.e., dendritic cells, monocytes, macrophages, microglia, and B cells) activate naïve CD4^+^ T cells and promote differentiation of CD4^+^ Th17 and Th1 cells through inflammatory cytokines (IL-1β, IL-6, and IL-23; and IL-12, respectively).

Activated microglia are rapidly recruited to sites of CNS damage (Duan et al., 2009). These APCs upregulate the expression of MHCII and other costimulatory molecules, such as CD40, CD80, and CD86 (Windhagen et al., 1995; Gerritse et al., 1996; Zrzavy et al., 2017). These observations suggest that activated APCs within the CNS possess the capacity to present antigens to infiltrating T cells. However, *in vitro* data show that microglia have a limited capacity to activate CD4^+^ T cells (Mack et al., 2003). In contrast to the *in vitro* findings, a dynamic alteration in the presence and phenotype of microglia within the CNS has been reported with regard to the absence or presence of lesions within the local microenvironment (Esiri and Reading, 1987; Ferguson et al., 1997; Prineas et al., 2001; Zrzavy et al., 2017). For example, microglia activation was more pronounced and increased with the length of disease even in the normal-appearing white matter sections from MS patients as compared to control tissue samples (Zrzavy et al., 2017). Additionally, active lesions from MS patients contained microglia and macrophages expressing a pro-inflammatory phenotype (Zrzavy et al., 2017), suggesting the ability of these cells to activate CD4^+^ T cells.

Besides microglia and macrophages, recent evidence suggests that B cells may serve as an important APC population in RRMS disease pathogenesis. To test this hypothesis, the ability of memory B cells from RRMS patients to activate CD4^+^ T cell in response to MBP and MOG was compared to naïve B cells from health donors. The data show that the naïve B cells from healthy donors did not activate the CD4^+^ T cells in the presence of MBP and MOG, while the memory B cells from RRMS patients did activate the CD4^+^ T cells (Harp et al., 2010). In the context of anti-CD20 therapy, which deletes peripheral B cells, the aforementioned findings suggest that the depletion of B cells following anti-CD20 treatment may be due in-part to the loss of B cells as an APC population. This possibility is supported by studies utilizing whole MOG protein-induced EAE in C57BL/6 mice, in which MOG-specific B cells are activated (Hausler et al., 2018).

**FIGURE 1:**
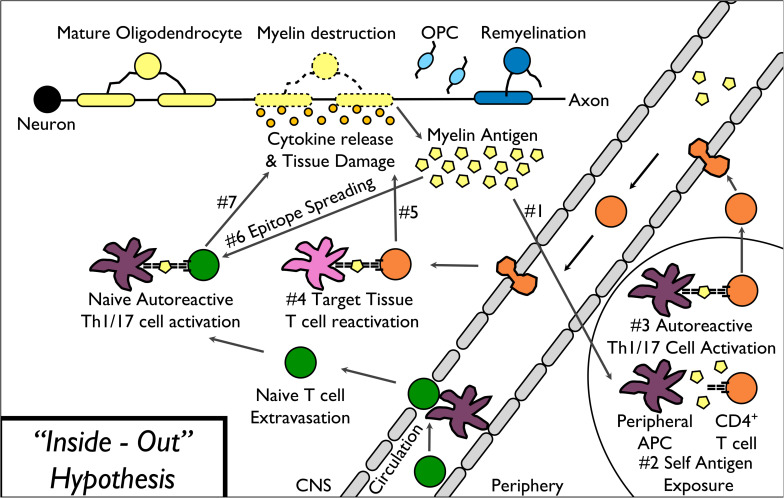
“Inside-Out” model of MS pathophysiology. The “inside-out” model of MS pathogenesis begins with the release of myelin antigens from injured or destabilized myelin to the periphery (1) followed by the presentation of myelin epitopes to (2) and activation of autoreactive T cells (3). Activated autoreactive T cells then migrate into the CNS, are reactivated by CNS-resident APCs (4), and release cytokines leading to direct as well as indirect damage to myelin (5). Additional myelin epitopes released by the primary T cell response induce epitope spreading (6) leading to additional myelin destruction (7).

**FIGURE 2:**
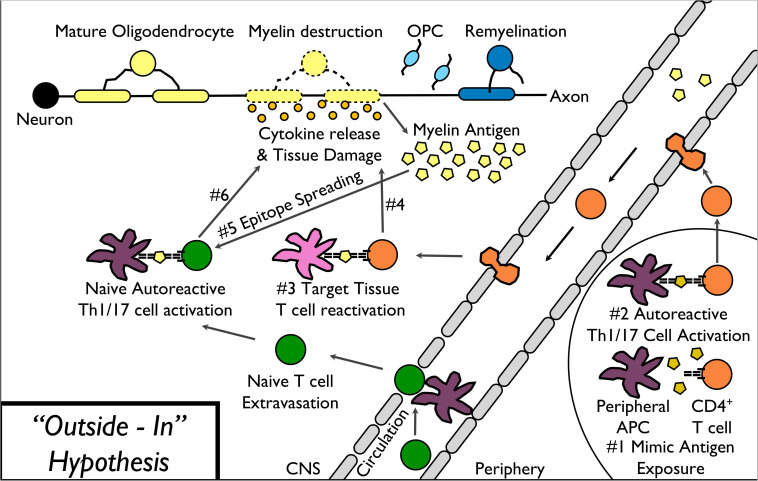
“Outside-In” model of MS pathophysiology. The “outside-in” model of MS pathogenesis begins with activation of myelin-specific T cells in response to a myelin peptide mimic epitope expressed on a pathogenic virus or other microbe exposure (1–2). Activated autoreactive T cells then migrate into the CNS, are reactivated by CNS-resident APCs (3), and release cytokines leading to direct as well as indirect damage to myelin (4). Additional myelin epitopes released by the primary T cell response induce epitope spreading (5) leading to additional myelin destruction (6).

In the “inside-out” model of MS (Figure 1), amplified inflammation, driven by peripherally derived autoreactive CD4^+^ Th17 and Th1 cells, directly and indirectly leads to further myelin destruction (Glass et al., 2010; Prinz and Kalinke, 2010). Based on the presence of T cell-mediated inflammation within the CNS of MS patients, the field historically utilizes “outside-in” experimental models of disease (Figure 2). These experimental models include relapsing-remitting experimental autoimmune encephalomyelitis (R-EAE) and Theiler’s murine encephalomyelitis virus (TMEV) infection in SJL/J mice, chronic experimental autoimmune encephalomyelitis (C-EAE) in C57BL/6 mice, and more recently a non-human primate model of virus-induced Japanese macaque encephalomyelitis (JME) (Table 2).

### Experimental Autoimmune Encephalomyelitis (EAE)

In mice, the MS disease processes, including myelination defects, axonal pathology, and immune cell infiltration can be experimentally recapitulated. Classically, the experimental autoimmune encephalomyelitis (EAE) mouse model has been used to mimic autoimmune demyelination in response to a peripheral immune-priming event serving as an “outside-in” approach. Both relapsing-remitting MS (RR-MS) and primary-progressive MS (PP-MS) can be arguably modeled by EAE induced via subcutaneous priming of different mouse strains with specific myelin peptides in complete Freund’s adjuvant (CFA). CD4^+^ Th1/17 cells primed in the peripheral lymph nodes, traffic to the CNS, and are re-stimulated with endogenous myelin antigens leading to effector responses and clinical disease. Priming SJL/J mice with proteolipid protein (PLP)_139__–__151_/CFA results in multiple clinical relapses (R-EAE) and priming C57BL/6 mice with myelin oligodendrocyte glycoprotein (MOG)_35__–__55_/CFA and pertussis toxin or infection of SJL/mice with TMEV induces an acute phase of disease followed by chronic progression (C-EAE) as measured by clinical scoring (Theiler and Gard, 1940; Veillette et al., 1989; Ben-Nun et al., 1991; Burns et al., 1991; Sun et al., 1991; Trotter et al., 1991; Soderstrom et al., 1993; Zhang et al., 1993; Steinman et al., 2002; Sospedra and Martin, 2005; Robinson et al., 2014; Terry et al., 2016). The use of the R-EAE and C-EAE mouse models allow assessment of motor function via clinical scoring, immune cell function, promotion of oligodendrocyte proliferation/maturation, and formation of new myelin.

Ideally, re-establishing self-tolerance would be induced by antigen-specific immune therapy (i.e. immune tolerance) in which the remaining immune system functions remain intact. The utilization of both R-EAE and C-EAE models of disease have been used to identify the underlying epitope-spreading mechanism within autoimmune disease. Epitope spreading in R-EAE has been clearly defined during the various phases of disease (Miller et al., 1995). During the immune response to a foreign or self-protein, the initial CD4^+^ T cell response focuses on one or two antigenic peptide epitopes within the immunogenic protein. These initial immunogenic epitope(s) are termed the dominant epitope(s). As the immune response progresses, the process of epitope spreading occurs, which is defined as the activation of additional antigen-specific CD4^+^ T cells that express T cell receptors specific for additional antigens that are not the dominant epitope(s) (Lehmann et al., 1992, 1993; Vanderlugt and Miller, 2002). For example, in an SJL/J mouse primed with PLP_139__–__151_/CFA, PLP_139__–__151_-specific CD4^+^ T cell reactivity is induced within 3 days of priming in the draining lymph nodes for the site of PLP_139__–__151_/CFA injection, and this dominant epitope-specific CD4^+^ T cell response is maintained throughout the disease course. Immediately before, and continuing during, the primary relapse phase of disease, PLP_178__–__191_ reactivity (termed intramolecular epitope spreading, i.e., spreading from one peptide epitope to another peptide epitope contained within the same protein) is detected by T cell proliferation and delayed-type hyper-sensitivity (DTH) assays. During the secondary relapse phase of disease, MBP_84__–__104_ responses (termed intermolecular epitope spreading, i.e., spreading from one peptide epitope to another peptide epitope contained within a different protein) are detectible. Conversely, if SJL/J mice are primed with PLP_178__–__191_/CFA, the acute phase of disease is mediated by CD4^+^ T cell responses to the initiating PLP_178__–__191_ epitope. Subsequently, PLP_139__–__151_ CD4^+^ T cells are detectible within the spleen and cervical lymph nodes during the primary disease relapse, and MBP_84__–__104_ specific CD4^+^ T cells during the secondary disease relapse. Published data show that while the detection of the spread epitope-specific CD4^+^ T cells (PLP_178__–__191_ or PLP_139__–__151_ specific CD4^+^ T cells depending on the peptide used to induce disease) does not occur until the primary disease relapse, these spread epitope-specific CD4^+^ T cells are initially activated during the acute phase of disease via antigen presenting cells presenting spread epitope peptides within the CNS (McMahon et al., 2005). Similarly, infection of SJL/J mice with TMEV, results in the bystander immune-mediated CNS damage leading to initial epitope spreading to PLP_139__–__151_ followed by responses to additional myelin epitopes. The development of these responses correlates with the extent of myelin destruction during the acute disease phase (McRae et al., 1995). The hierarchy of dominant epitopes is due to a combination of differential protein processing and presentation by various APCs, and the precursor frequency of the antigen-specific CD4^+^ T cells (Lehmann et al., 1998; Moon et al., 2007).

The epitope spreading phenomena during autoimmune disease has been confirmed by the use of antigen-specific tolerance therapies. For example, immune tolerance is readily induced by coupling of peptides to donor splenocytes (SP) using the chemical crosslinker 1-ethyl-3-(3- dimethylaminopropyl) carbodiimide (ECDI) (Wetzig et al., 1979). Antigen coupled to splenocytes (Ag-SP) delivers the antigen to APCs that present the cargo antigen in a tolerogenic manner. The non-specific crosslinking of antigen to the cell surface while inducing apoptosis allows the donor cells to be perceived by the host in a non-inflammatory (non-immunogenic) manner. Ag-SP have been employed to prevent and treat the relapsing EAE model of MS (Podojil and Miller, 2009), and type 1 diabetes (T1D) in the non-obese diabetic (NOD) mouse (Prasad et al., 2012). A recent publication summarized the results of a phase I trial in MS patients using apoptotic ECDI-fixed peripheral blood mononuclear cells (PBMCs) pulsed with a cocktail of myelin peptides, illustrating the safety and efficacy of this procedure in human autoimmune disease (Lutterotti et al., 2013). Importantly, the mechanistic aspects of this study provided an important proof-of-principle that induced peripheral tolerance can be successfully employed to induce unresponsiveness in human autoreactive T cells.

More recently, biodegradable carboxylated nanoparticles composed of poly(lactic-*co*-glycolic) acid (PLGA) were shown to induce antigen-specific tolerance for prevention and treatment of EAE when encephalitogenic peptides were EDCI fixed to the surface of the particles or encapsulated within the particles (Getts et al., 2012; Hunter et al., 2014). Administration of Ag-bearing PLGA nanoparticles results in significantly reduced CNS infiltration of encephalitogenic Th1 (IFN-γ) and Th17 (IL-17a and GM-CSF) cells as well as inflammatory monocytes/MΦs. Tolerance was most effectively induced by intravenous infusion of Ag-PLG (Getts et al., 2012), though intraperitoneal delivery was also able to attenuate disease scores. The intravenous route likely has greater efficacy due to direct trafficking and uptake of the nanoparticles by APCs in the liver and spleen via the macrophage receptor of collagenous structure (MARCO) scavenger receptor (Getts et al., 2011).

### Theiler’s Murine Encephalomyelitis Virus-Induced Demyelinating Disease (TMEV-IDD)

As outlined above, we have previously extensively studied and reviewed (Croxford et al., 2002; Munz et al., 2009) the immunopathogenesis of TMEV-induced demyelinating disease (TMEV-IDD) “outside-in” model of MS. Briefly, TMEV is a picornavirus which naturally enters the CNS via a fecal-oral transmission route and enters the CNS via a retrograde transport mechanism. In experimental TMEV-IDD, disease is induced by intracerebral injection of TMEV which then induces a persist infection of microglia which, in susceptible mouse strains, stimulates inflammatory anti-viral immune responses (Th1, Th17, and CD8) which cause bystander damage to oligodendrocytes and myelin in the CNS. Released myelin antigens then activate myelin epitope-specific autoimmune responses via the process of epitope spreading (Miller et al., 1997) leading to a chronic demyelinating and a spastic course of paralysis. We also showed that a strain of TMEV engineered to express molecular mimic of the myelin PLP_139__–__151_ epitope sharing only 3 of the 13 amino acid residues (critically including the primary MHC binding and the primary and secondary T cell receptor binding residues), could induce demyelinating disease via the process of molecular mimicry (Olson et al., 2001). Collectively these studies indicate that myelin-specific autoimmune pathology can be induced by infection both via bystander damage induced release of self-antigens (epitope spreading) and molecular mimicry.

### Japanese Macaque Encephalomyelitis (JME)

Japanese macaque encephalomyelitis (JME) is an inflammatory demyelinating disease that occurs spontaneously in a colony of Japanese macaques (JM) at the Oregon National Primate Research Center (Axthelm et al., 2011; Blair et al., 2016). The disease only occurs in specific lineages within the colony, and is triggered by a novel gamma-herpes virus, Japanese macaque rhadinovirus (JMRV), that occurs spontaneously in 1–3% of the JM colony and with targeted breeding 2–5% (Axthelm et al., 2011). If needed, based on population and time constraints, disease can be induced by intracranial injections of JMRV into animals from affected lineages (Estep et al., 2013) with the advantage of a known consistent location for histology and MRI/DTI. Animals with JME display clinical signs resembling Multiple Sclerosis, such as; ataxia, paresis, and magnetic resonance imaging reveals multiple T2-weighted hyperintensities and gadolinium-enhancing lesions in the central nervous system (i.e., brainstem, cerebellum, and cervical spinal cord). The prevalent myelin epitope is myelin basic protein (MBP). Comparable to disease manifestation in MS patients, the CNS of animals with JME present with active lesions that contain CD4^+^ Th1 and Th17 cells, CD8^+^ T cells, and oligoclonal bands are present within the CSF (Blair et al., 2016).

In addition to testing immune modulatory therapies, therapies that potentially promote myelin repair by stimulating oligodendrocyte progenitor cell expansion, homing and/or differentiation can be assessed in the EAE and TMEV-IDD mouse and JME primate models of MS. Researchers can examine clinical disease progression in the form of sensory and motor function, CNS immune and inflammatory responses, flow cytometry-based enumeration of cells of the oligodendrocyte lineage, and changes in myelin. The use of the MS-like animal disease models provides a robust platform for assessing combined immune regulation and myelin repair therapies in an “outside-in” model of CNS immune-induced demyelinating disease. These *in vivo* platforms for testing new myelin repair drugs will hopefully lead to the translation of a novel drug, either alone or in combination with immune regulatory drugs, for the treatment of MS.

## “Inside-Out” MS Models

The “inside-out” model proposed by Stys et al. (2012) argues that primary degeneration of oligodendrocytes and myelin is the initial event of MS, and might occur in the earliest years before the onset of symptoms. Primary oligodendrocyte death and/or subtle myelinopathy can precede and subsequently drive a secondary autoimmune attack, resulting in inflammatory demyelination in MS (Figure 1). Therefore, there has been a search for agents that could trigger these CNS events, resulting in the onset of an immune response to myelin.

### Epsilon Toxin Model

In the earliest stage of MS, the histologic description of formation of nascent lesions without inflammatory infiltration argues for the possibility of an “inside-out” mechanism (Barnett and Prineas, 2004; Prineas and Parratt, 2012). Observation of oligodendrocyte apoptosis along with blood brain barrier disruption in the nascent lesions indicates that MS might arise from an environmental insult targeting oligodendrocytes, such as a toxin or virus. Epsilon toxin is produced by the type B and the type D strains of *Clostridium perfringens*, a spore-forming gram-positive bacterium mostly found in the intestines of ruminant animals (Blackwell et al., 1983; Uzal et al., 2004; Uzal and Songer, 2008). Epsilon toxin is converted into an active form, crosses the intestinal mucosa and disseminates via the bloodstream, massively accumulating in the brain and kidneys (Finnie, 1984, 2003; Tamai et al., 2003). The toxin has the capability to cross the blood brain barrier and infiltrate the brain parenchyma, which results in MS-like symptoms (Dorca-Arevalo et al., 2008; Popoff, 2011). Over three decades ago, Murrell et al. (1986) first hypothesized that epsilon toxin is the potential toxin that triggers MS, even though humans are not natural hosts for *C. perfringens* types B and D. More recently, *C. perfringens* type B was isolated from the stool of a female remitting-relapsing MS patient with an onset 3 months previously (Rumah et al., 2013). Furthermore, epsilon toxin specific antibodies were found in serum and/or CSF of 10% of MS patients and 1% of healthy controls from the banked samples in the United States (Rumah et al., 2013). Similarly, immunoreactivity toward epsilon toxin in serum is higher in MS patients than in controls in the United Kingdom (Wagley et al., 2019). In light of these observations and clinical evidence, it has been proposed that epsilon toxin exposure may play a role in initiating MS lesion formation by binding to oligodendrocytes, myelin and white matter (Lonchamp et al., 2010; Wioland et al., 2015). Although the mechanism underlying the effect of epsilon toxin on oligodendrocytes and subsequent demyelination is not yet clear, several lines of evidence *in vitro* indicate that epsilon toxin selectively attacks mature oligodendrocytes and triggers demyelination (Linden et al., 2015; Wioland et al., 2015; Bossu et al., 2020). It has been shown that myelin and lymphocyte protein (MAL) could be a candidate epsilon toxin receptor on oligodendrocytes (Rumah et al., 2015). Once bound to oligodendrocytes, epsilon toxin could lead to the rise of glutamate and subsequent activation of mGluR, which activates intracellular Ca^2+^signaling and eventually triggers demyelination (Lonchamp et al., 2010; Kostic et al., 2013; Wioland et al., 2015). In line with this evidence, it is highly possible that an agent cytotoxic to oligodendrocytes may trigger MS.

### Diphtheria Toxin A Chain (DTA) Model

One of the proposed factors in the alternative “inside-out” theory for initiating MS is the primary cytotoxic death of oligodendrocytes. A toxin-induced ablation of oligodendrocytes is useful for testing whether such oligodendrocyte death could trigger anti-CNS autoimmunity (Jakel and Dimou, 2017). *Plp1-CreER^T^; ROSA26-eGFP-DTA* (DTA) is a mouse model of oligodendrocyte ablation accomplished via an oligodendrocyte specific activation of toxin expression in adult mice. The A subunit of diphtheria toxin (*DT-A*) induces cell death by catalyzing the inactivation of elongation factor 2, thereby halting global protein synthesis (Collier, 2001). The *Plp1-CreER^T^* mouse line drives expression of the tamoxifen-regulated Cre recombinase under control of the oligodendrocyte-specific myelin proteolipid protein (PLP) transcriptional control region (Doerflinger et al., 2003). The *DT-A* expression in oligodendrocytes is the result of tamoxifen-induced Cre-mediated recombination of *ROSA26-eGFP-DTA* locus via *Plp1-CreER^T^* (Traka et al., 2010). The expression of the *DT-A* subunit specifically in CNS myelinating oligodendrocytes results in widespread oligodendrocyte ablation, CNS demyelination, and the subsequent development of severe neurological symptoms. There is no breakdown of the blood brain barrier or detectible increase in CD4^+^ T cell into the CNS despite local inflammation, and there is no apparent loss of CNS axons during the initial demyelination event. However, the specific phenotype(s) of the T cells within the CNS during this initial demyelination phase of disease have not been fully characterized. The clinical symptoms of the acute phase of the disease are ameliorated during a recovery phase that correlates with the repopulation of mature oligodendrocytes and robust remyelination in the following 6–7 weeks (Traka et al., 2010). Interestingly, as the recovered mice age, they develop a secondary lethal progressive demyelinating disease starting around 40 weeks after tamoxifen injection that is mediated by MOG_35__–__55_-specific CD4^+^ T cell infiltration into the CNS during the late stages of disease (Traka et al., 2016). While the MOG_35__–__55_-specific CD4^+^ T cell responses are detectable in the peripheral lymphoid organs at 40 weeks post induction and are not present at 10 weeks, there is a significant increased number of CD4^+^ T cells in the CNS at 10 weeks (Traka et al., 2016). This increase in CNS CD4^+^ T cells may correlate with the expansion of myelin-specific T cells, similar to the initial activation and expansion of spread-epitope-specific CD4^+^ T cells within the CNS in EAE (McMahon et al., 2005; Bailey et al., 2007). The late-stage pathology is due to the induction of CD4^+^ T cell-mediated autoimmune responses secondary to oligodendrocyte ablation via a non-immune-mediated event (i.e. DT-mediated toxicitiy). This is supported by the major findings that adoptive transfer of the myelin-specific CD4^+^ T cells derived from DTA mice into naïve mice consistently results in the induction of mild neurological symptoms and inflammatory CNS lesions in the recipients, and induction of immune tolerance using the MOG_35__–__55_-coupled PLG nanoparticles significantly inhibits the progression of late-onset disease symptoms in DTA mice protecting animals from eventual fatal demyelinating disease (Traka et al., 2016).

In addition to the DTA mouse model, other genetic mouse models were later developed to achieve a faster oligodendrocyte ablation via expressing diphtheria-toxin receptor (DTR) under the MOG-promoter accompanied by direct administration of diphtheria toxin (Ghosh et al., 2011; Locatelli et al., 2012; Oluich et al., 2012; Gritsch et al., 2014). However, some studies reported that a secondary anti-CNS immunity did not develop in these mice, that is most likely due to premature death of these mice (Locatelli et al., 2012; Gritsch et al., 2014). The development of CNS immunity after oligodendrocyte death appears to be a slow process, taking several months in the DTA mouse model described above.

The DTA mouse model supports the “inside-out” theory, recapitulating pathological evidence showing that the loss of oligodendrocytes and subsequent demyelination may result in the induction of autoreactivity against myelin antigens as well as secondarily lead to inflammation and demyelination in the CNS. The unique DTA mouse model system allows fundamental unanswered questions concerning the molecular and cellular mechanisms associated with the induction of the autoimmune response to contribute to the understanding of MS disease pathogenesis and to the development and testing of remyelination therapies.

### Cuprizone Autoimmune Encephalitis (CAE) Model

In addition to diphtheria-toxin, cuprizone is a demyelinating neurotoxin that has been used in testing “inside-out” hypothesis. Long-term of cuprizone feeding in mice lead to oligodendrocyte death, demyelination and gliosis (Matsushima and Morell, 2001; Sen et al., 2019b). Unlike DTA model, cuprizone feeding did not evoke a peripheral immune response in the CNS (Caprariello et al., 2018; Sen et al., 2019a). Some studies reported that the failure of cuprizone feeding to trigger in triggering CNS immune response is due to the atrophy of immune organs like the spleen and thymus (Solti et al., 2015; Partridge et al., 2016; Sen et al., 2019a). A more recent study reported that cuprizone induced demyelination can trigger an “inside-out” immune response when the BBB is disrupted by pertussis toxin (Almuslehi et al., 2020).

Accumulating clinical evidence suggests that primary myelin destabilization by citrullination releases immunogenic myelin debris and subsequently drives a secondary autoimmune attack (Moscarello et al., 1994; Cao et al., 1999; Stys et al., 2012). Excessive citrullination of myelin basic protein (MBP) had been found in normal appearing white matter from postmortem MS brain tissues and the extent of modified myelin is related to the severity of MS (Moscarello et al., 1994; Wood et al., 1996; Bradford et al., 2014). Citrullination is a post-translational modification mediated by peptidylarginine deiminase (PAD). Citrullination occurs when a positively charged arginine residue is deiminated to a neutrally charged citrulline (Vossenaar et al., 2003; Moscarello et al., 2007). Due to the changed charge in the protein, citrullinated MBP is partially unfolded and cannot stabilize a compact myelin sheath (Beniac et al., 2000; Bakhti et al., 2013). Studies have shown that deiminated MBP with citrulline is more susceptible to proteolytic digestion and that the digestion rate is remarkably correlated with the amount of citrulline present in MBP peptides (Cao et al., 1999; D’Souza and Moscarello, 2006; Musse et al., 2006). Increased breakdown of citrullinated MBP results in generating immunodominant epitopes (Musse et al., 2006); potentially triggering autoimmunity and eliciting destructive inflammatory demyelination (Raijmakers et al., 2005).

A newly developed mouse model of cuprizone autoimmune encephalitis (CAE) provides direct evidence to support the causative relationship between primary abnormalities of myelin and inflammation (Caprariello et al., 2018), whereby biochemical destabilizing myelin triggers a secondary inflammatory demyelination comparable to active MS lesions. The CAE is initiated with a 2-week exposure of neurotoxicant cuprizone to perturb myelin without causing overt demyelination, followed by an immune boost of complete Freund’s adjuvant (without exogenous antigen) and pertussis toxin. After 2 weeks, these mice develop inflammatory demyelination which resembles pathology found in MS patients and the EAE mouse model (Caprariello et al., 2018). The histopathological changes of the CAE model are characterized by periventricular and white matter tract gadolinium enhancement of MRI of the brain as well as overt demyelination and cellular infiltration within the corpus callosum. Gadolinium enhancement indicates breakdown of the BBB as a result of active inflammation. However, removal of the immune boost abrogates these responses, implying the importance of an immune-permissive environment. Most importantly, suppression of the destructive immune response by administration of peptidyl arginine deiminase (PAD) inhibitors to the CAE mice suggests that citrullinated proteins altered by abbreviated cuprizone exposure possibly drive the inflammatory demyelinated lesions in CAE.

### Additional “Inside-Out” Models

Although genetic mutations of myelin proteins as well as traumatic brain injury-associated dysmyelination have been linked to later development of MS, it is not yet clear what initially triggers citrullination of myelin proteins or subtle dysmyelination (Warshawsky et al., 2005; Donovan et al., 2014; Sidaway, 2017; Cloake et al., 2018).

Adrenoleukodystrophy (ALD) is an X-linked neurometabolic disorder due to mutations in a proximal transporter, adenosine triphosphate (ATP)-binding cassette, subfamily, member 1 gene (*ABCD1*) (Moser et al., 2007; Kemp et al., 2016). The clinical presentation of ALD is complex; involving adrenal insufficiency and myelopathy (de Beer et al., 2014; Kemp et al., 2016). Approximately 60% of male patients develop rapidly progressive inflammatory cerebral demyelination (Moser et al., 1992), which clinically coincides with a progressive neurological decline similar to MS (Ferrer et al., 2010; Brandao de Paiva et al., 2018). However, the complex mechanisms on how this metabolic disease is transitioned to a fatal neuroinflammatory disease remains elusive. *ABCD1* mutation may prevent transport of very long-chain fatty acids (VLCFAs) into peroxisomes for oxidation and degradation (Moser et al., 2007; Kemp et al., 2016). Some studies suggest that the accumulation of VLCFAs in myelin could mediate myelin instability and initial demyelination, which are believed to contribute to initiation of the inflammatory disease (Ho et al., 1995; Ito et al., 2001; Singh et al., 2009). The findings of CD1 (antigen presenting molecule) -mediated lipid antigen presentation in cerebral ALD lesions supported the hypothesis that VLCFA-containing proteolipid protein in myelin may be a potential lipid antigen for triggering autoimmunity after myelin breakdown (Ito et al., 2001). The lesions progress rapidly accompanied by the opening of blood brain barrier and invasions of inflammatory cells (Powers et al., 1992; Ito et al., 2001). Further evidence for the involvement of different components of the immune system in the pathogenesis of demyelinating ALD was reviewed in Hudspeth and Raymond (2007). Interestingly, the demyelinating progress arrested in a small percentage of patients with initial cerebral demyelination, in which the disruption of the blood brain barrier does not occur (Korenke et al., 1996). The importance of blood brain barrier in ALD was emphasized by several pieces of evidence that suggested potential environmental factors, such as head trauma (Weller et al., 1992; Raymond et al., 2010), possibly increase permeability of the blood brain barrier, which either trigger or precipitate the demyelination.

Traumatic brain injury (TBI) is most commonly caused by an external head impact that injures the brain. Demyelination and irreversible axon damage, particular in the corpus callosum, represent major pathological features frequently observed in TBI patients (Rutgers et al., 2008; Armstrong et al., 2016a; Chung et al., 2018; O’Phelan et al., 2018). Nevertheless, the progression of white matter injury is poorly understood in TBI. Oligodendrocytes are known to be vulnerable to oxidative stress and excitotoxicity following traumatic injury (Lotocki et al., 2011; Giacci and Fitzgerald, 2018). The loss of oligodendrocytes could significantly contribute to underlying demyelination after injury (Dent et al., 2015; Armstrong et al., 2016b) and activate neuroinflammation (Mierzwa et al., 2015). Furthermore, the observation that a persistent adaptive immune response in the CNS developed in the mice weeks after TBI (Daglas et al., 2019) fits within the “inside-out” theory. Interestingly, neuroinflammation, which persists for years after TBI, has recently been shown to largely contribute to neurodegeneration and long-term neurological dysfunction (Bazarian et al., 2009; Amor et al., 2010; Daglas et al., 2019). In particular, genetic depletion of CD8+ T cells in TBI mouse model improves neurological outcomes (Daglas et al., 2019), which indicates the importance of neuroinflammation in the progression of TBI. Encouragingly, damping neuroinflammation with immunomodulatory nanoparticles results in reduced neuropathology and neurophysiological abnormalities following TBI, suggesting a potential therapeutic strategy (Sharma et al., 2020).

Pre-clinical and clinical findings in ALD as well as TBI could shed light on potential MS therapeutics and vice versa. Current MS treatments are mainly directed to immune suppression, but the CAE model provides evidence for a potential “inside-out” mechanism of initiation of chronic demyelination and could serve as a compelling preclinical model of MS translational studies for the development of myelin-protective strategies in early stages of the disease.

## Relevance for the Clinic

As we have highlighted the “outside-in” (Figure 2) peripheral immune driven models and “inside-out” (Figure 1) neurodegenerative models (Table 2), the next section will focus on the relevance for the clinic. The “outside-in” pathogenesis (Figure 2) begins with activation of myelin-specific T cells in response to a myelin peptide mimic epitope expressed on a pathogenic virus or other microbe exposure. Activated autoreactive T cells then migrate into the CNS, are reactivated by CNS-resident APCs, and release cytokines leading to direct as well as indirect damage to myelin. Additional myelin epitopes released by the primary T cell response induce epitope spreading, leading to additional myelin destruction. During repair, a mild inflammatory reaction can stimulate oligodendrocyte precursor cells and “protective autoimmunity” utilizing T regulatory cells (Schwartz and Raposo, 2014). Unfortunately, the eventual failure of myelin repair during RRMS leads to chronically demyelinated axons, which degenerate over time and contribute to disease progression (Franklin and Ffrench-Constant, 2008; Trapp and Nave, 2008). Neuronal injury occurs, in part, as a result of inflammation mediated by myelin-specific CD4^+^ T cells. Direct and indirect effects of neuroantigen-specific Th1/17 cells can lead to demyelination and subsequent neuronal dysfunction by mechanisms that include activation of microglia and infiltrating inflammatory monocytes/macrophages by pro-inflammatory cytokines (IFN-γ, IL-17, and GM-CSF), which then produce proteases and additional pro-inflammatory cytokines, nitric oxide (NO) and reactive oxygen species (ROS), which induce myelin and axonal damage (Glass et al., 2010). Neuronal loss is thought to be a consequence of the demyelination, which causes dramatic ionic and energy imbalances in axons resulting from the loss of the structural and trophic support provided by oligodendrocytes (Trapp and Nave, 2008; Nave, 2010). This subsequent “inside-out” pathogenesis (Figure 1) can then spread and lead to further loss of axonal integrity. This perpetuates the release of myelin antigens to the periphery followed by the presentation of myelin epitopes to and activation of autoreactive T cells; ultimately leading to the progressive diffuse atrophy of the brain.

### MS Patient Therapeutics

Disease modifying therapies for MS include immunomodulatory and immunosuppressive medications that suppress or modulate the self-reactive immune responses. While most of the medications exert their effects in the peripheral immune organs or blood stream, some also have the capacity to modulate the local immune responses and oligodendrocytes in the CNS.

In patients, Dimethyl fumarate (Tecfidera) decreased B cell CD40 expression (disrupted B-cell activation), decreased memory T cells, and decreased T cell proliferation and activation; resulting in lymphopenia (Linker and Gold, 2013). The mechanism of action of dimethyl fumarate within the CNS involves both Nrf-2-dependent as well as independent pathways for neuroprotection involving diminished neuroinflammation (Mills et al., 2018; Yadav et al., 2019). The Nrf2-dependent pathway promotes neuroprotection, oligodendrocyte survival, and decreases astrocyte activation (Linker et al., 2011; Kalinin et al., 2013; Wang et al., 2015; Zarrouk et al., 2017). The Nrf2-independent pathway also increases neuroprotection and decreases astrocyte activation, specifically reactive oxygen species production (i.e., Nitric Oxide) (Lin et al., 2011). Additionally, dimethyl fumarate targets innate immunity, in the form of microglia, resulting in diminished activation by the Nrf2 independent pathway (Parodi et al., 2015).

In patients, Fingolimod (Gilenya) suppresses migration of peripheral lymphocytes (Brinkmann et al., 2010; Francis et al., 2014). The mechanism of action of Fingolimod is the modulation of S1P receptor expression, most notably S1P1 receptor associated with lymphocytes, diminishing the number of T cells infiltrating into the CNS by retaining T cells in the lymph nodes (Brinkmann et al., 2002; Mandala et al., 2002; Fujino et al., 2003; Pham et al., 2008). Additionally, Fingolimod is neuroprotective, functioning within the CNS on neurons (Balatoni et al., 2007; Lee et al., 2010), oligodendrocyte lineage cells (Zhang et al., 2015), and decreasing the hyperactivity of reactive astrocytes (Choi et al., 2011). Of note, cumulatively Fingolimod has been shown to improve white matter integrity in relapsing remitting MS patients (Gurevich et al., 2018). Fingolimod can have “off target” effects as it can interact with multiple S1PR subtypes (S1PR1, S1PR3, S1PR4, and S1PR5) in a variety of tissues, including the heart (Chaudhry et al., 2017). The field has shifted to developing new therapies to mitigate these side effects, that selectively target subtype 1 of S1PR, yet this may diminish the neuroprotective capacity and immune suppression as S1PR5 is associated with oligodendrocyte function and natural killer cells (Chaudhry et al., 2017).

Natalizumab (Tysabri) is a monoclonal antibody against very late activating antigen (VLA)-α4 integrin and can bind to a majority of leukocytes, impeding cross over through the blood brain barrier into the CNS, thereby diminishing the aberrant heightened immune surveillance and inflammation (Stuve et al., 2006). However, use of Natalizumab in patients infected with John Cunningham virus (JCV) can result in progressive multifocal leukoencephalopathy (PML) in a subset of patients (Clerico et al., 2017; Ho et al., 2017; Fragoso et al., 2019; Ryerson et al., 2019). JCV is an opportunistic virus causing oligodendrocyte destruction, demyelination, and eventually a detrimental inflammatory reaction. Natalizumab associated PML, leading to subsequent CNS inflammation and worsening of MS, underscores the interplay between the generation of free antigen (viral and myelin), T cell immune surveillance, and the rebalancing mechanisms of neuroinflammation. Overall, despite vast strides in disease modifying therapy (DMT) options for MS patients over the last few decades, substantial risk for adverse side effects remain with the existing therapies.

## Discussion/Conclusion

As Multiple Sclerosis is a syndrome with multiple clinical presentations and not a single disease entity, it is likely that both immune (outside-in) and neurodegenerative (inside-out) driven molecular pathways can initiate the etiopathogenesis of MS in different patients (Table 1). Additionally, it is important to consider that these two mechanisms are not mutually exclusive as, regardless of the initiating event, both Immune-mediated and neurodegenerative processes are important components of both types of models with the difference being the timing of the two processes. Each highlighted model has benefits yet limitations and not all pre-clinical models of MS were covered (Lassmann and Bradl, 2017). We highlighted both conventional as well as new experimental models for testing novel MS therapeutics, while exploring the underlying role of the adaptive and innate immune systems (Table 2). The three therapeutic targets for balancing immune dysfunction and preventing neurodegeneration necessary for effective amelioration of MS progression include: re-establishing self-tolerance, neuroprotection, and promotion of remyelination.

Chronic immunosuppression and immunomodulation are the most commonly used therapeutic strategies for MS, outside of symptom management. In addition to traditional disease-modifying therapies, immune reconstitution therapy (IRT) has emerged as a novel treatment paradigm (AlSharoqi et al., 2020; Derfuss et al., 2020). The latter is based on partial or full ablation of the immune system aiming to destroy self-reactive clones and restore normal function. Though attractive in principle, immune reconstitution at present is an uncontrolled process whose long-term efficacy and side effects remain to be established. Furthermore, IRT is a costly therapy that is available only in certain medical centers and typically reserved for patients with highly active disease. Immunotherapy based on re-establishing of self tolerance is likely to be more advantageous to patients in terms of disease control and avoidance of immunosuppressive side effects. Such strategy also may set the basis for personalized therapies of MS, where patient specific autoimmune responses are targeted by tolerizing agents. The Miller lab has recently demonstrated an effective means of ameliorating ongoing disease in EAE mouse models of MS by inducing tolerance in autoreactive CD4^+^ T cells using intravenous (i.v.) infusion of 500 nM poly(lactic-*co*-glycolic acid) nanoparticles coupled with or encapsulating myelin peptides (Ag-PLG) that effectively reduces disease burden in relapsing-remitting (R-EAE) and in chronic-progressive (C-EAE) mouse models of experimental autoimmune encephalomyelitis (EAE) by reducing inflammatory cell activation and pro-inflammatory Th1/17 cytokine production (Getts et al., 2012, 2013; Hunter et al., 2014; McCarthy et al., 2017). Using myelin peptide-coupled autologous apoptotic leukocytes, we had previously demonstrated successful tolerance induction in MS patients (Lutterotti et al., 2013). Clinical testing of the Ag-PLG tolerance platform will be initiated in MS patients within the next year. We have recently shown, in a phase 1/2a trial in human celiac disease, safety and efficacy of PLG nanoparticles encapsulating gliadin (Kelly et al., 2019).

Interestingly, a recent single cell transcriptome study of oligodendrocyte lineage cells from the spinal cord of EAE mice indicated that oligodendrocytes and OPCs may not be passive targets of the immune attack, but rather involved in antigen-processing and presentation during the development of MS (Falcao et al., 2018). This possibility is supported by a previous study demonstrating that IFN-γ stimulated the production of chemokines from oligodendrocytes, while transgenic mice that suppresses oligodendrocyte responsiveness to IFN-γ developed an accelerated EAE onset (Balabanov et al., 2007).

The Popko lab has worked to enhance the protection of oligodendrocytes and myelin by augmenting the integrated stress response (ISR), a mechanism that protects endangered cells from inflammatory insults. Using a variety of mouse models of inflammatory demyelination, they have shown that genetic manipulations that compromise the ISR increase the susceptibility of oligodendrocytes in response to CNS inflammation (Lin et al., 2005, 2007) and that the genetic enhancement of the ISR, in contrast, provides increased protection to oligodendrocytes (Lin et al., 2008, 2013). Encouragingly, it has been shown that the ISR modulators, guanabenz and Sephin1, are able to protect oligodendrocytes against inflammatory stress through enhancing the ISR in MS mouse models (Way et al., 2015; Chen et al., 2019). Based on the “inside-out” theory, oligodendrocyte protection diminishes demyelination and reduces the generation of myelin debris, which likely decreases the exposure of myelin fragments and limits the autoimmune response. The success of these studies attests to the potential of oligodendrocyte protective therapeutics in MS.

At present, there are not any FDA approved therapies approved for myelin repair in MS despite successful pre-clinical trials. Utilizing both “outside-in” and “inside-out” models allows a comprehensive study of the multi-directional feedback between the CNS and periphery. As both immune dysregulation as well as inflammatory demyelination and neurodegeneration lead to disease progression, the field will need both “outside-in” and “inside-out” models to test single and combination therapies. Our collective goal as a field, of clinicians and scientists, is to improve patient outcomes and quality of life for those living with Multiple Sclerosis. In summary, the availability and utilization of these diverse models allows the MS field a robust platform for developing novel therapeutics targeting the autoimmune response, neuronal stress and promoting myelin repair.

## Author Contributions

HET, YC, JP, RB, BP, and SDM conceived and outlined the manuscript and edited the manuscript. HET and YC wrote the manuscript. AR, HET, and SDM contributed to figures preparation. All authors contributed to the article and approved the submitted version.

## Conflict of Interest

SDM is an academic co-founder, scientific advisory board member, paid consultant, and grantee of Cour Pharmaceutical Development Company, Inc; scientific advisory board member and grantee of NextCure, Inc., scientific advisory board member of Takeda Pharmaceutical Company and Myeloid Therapeutics. RB has received honorariums and research support from Biogen, Sanofi Genzyme, Genentech, and Alexion Pharmaceuticals, Inc. JP was employed by company Cour Pharmaceutical Development Company, Inc. The remaining authors declare that the research was conducted in the absence of any commercial or financial relationships that could be construed as a potential conflict of interest.
